# Influence of dairy by-product waste milk on the microbiomes of different gastrointestinal tract components in pre-weaned dairy calves

**DOI:** 10.1038/srep42689

**Published:** 2017-03-10

**Authors:** Y. F. Deng, Y. J. Wang, Y. Zou, A. Azarfar, X. L. Wei, S. K. Ji, J. Zhang, Z. H. Wu, S. X. Wang, S. Z. Dong, Y. Xu, D. F. Shao, J. X. Xiao, K. L. Yang, Z. J. Cao, S. L. Li

**Affiliations:** 1 State Key Laboratory of Animal Nutrition, Beijing Engineering Technology Research Center of Raw Milk Quality and Safety Control, College of Animal Science and Technology, China Agricultural University, Beijing 100193, P. R. China; 2 Department of Animal Science, Faculty of Agriculture, Lorestan University, PO Box 465, Khorramabad, Iran; 3 Sichuan Animal Science Academy, Animal Breeding and Genetics key Laboratory of Sichuan Province, Chengdu 610066, P. R. China; 4 Beijing Computing Center, Beijing 100094, P. R. China; 5 College of Animal Science, Xinjiang Agricultural University, Wulumuqi 830052, P. R. China

## Abstract

The community structure of colonised bacteria in the gastrointestinal tracts (GITs) of pre-weaned calves is affected by extrinsic factors, such as the genetics and diet of the calves; however, the dietary impact is not fully understood and warrants further research. Our study revealed that a total of 6, 5, 2 and 10 bacterial genera showed biologically significant differences in the GITs of pre-weaned calves fed four waste-milk diets: acidified waste milk, pasteurised waste milk, untreated bulk milk, and untreated waste milk, respectively. Specifically, generic biomarkers were observed in the rumen (e.g., *Bifidobacterium, Parabacteroides, Fibrobacter, Clostridium*, etc.), caecum (e.g., *Faecalibacterium, Oxalobacter, Odoribacter*, etc.) and colon (e.g., *Megamonas, Comamonas, Stenotrophomonas*, etc.) but not in the faeces. In addition, the predicted metabolic pathways showed that the expression of genes related to metabolic diseases was increased in the calves fed untreated waste milk, which indicated that untreated waste milk is not a suitable liquid diet for pre-weaned calves. This is the first study to demonstrate how different types of waste milk fed to pre-weaned calves affect the community structure of colonised bacteria, and the results may provide insights for the intentional adjustment of diets and gastrointestinal bacterial communities.

Waste milk is a major by-product of the dairy industry and includes low-quality colostrum, transitional milk, milk from cows administered veterinary drugs for the treatment of mastitis and/or other diseases, milk with high somatic cell counts, and unqualified commodity milk. The amount of waste milk accounts for approximately 2–4% of the total milk production and corresponds to 0.8–1.6 million tons per year in China. The large amount of discarded waste milk generates environmental pollution and represents the loss of a valuable resource that may serve as a good feed source for dairy calves because of its bulk quantities and high nutrient content. However, using waste milk for feeding calves is disputed, especially because of its potential pathogen load and antimicrobial agent content.

Potential pathogens may expose new-born calves to infectious diseases and harmful endotoxins. Thus, several methods of processing waste milk have been proposed to resolve issues regarding the potential pathogenesis of this product. Although pasteurisation has been popularly applied to treat waste milk to inactivate pathogenic bacteria, it has either little or no effect on spores^1^,^2^,^3^, protozoa^4^, most viruses^5^, *Mycobacterium avium subspeciesparatuberculosis*^6^, and some bacteria^7^. Nevertheless, pasteurisation leads to a massive reduction (4–7 log_10_) of pathogens^8^,^9^,^10^. Furthermore, feeding pasteurised waste milk to calves has been reported to result in better performance, health, and additional economic profit compared with feeding untreated waste milk^11^. Acidification is another popular method of processing waste milk, which reduces calf exposure to pathogenic bacteria in waste milk. In Finland and other Nordic and European countries as well as Canada and certain states in the USA, producers usually feed calves with waste milk preserved with formic acid^12^,^13^. Preserving waste milk by acidification inhibits the growth of or kills pathogenic bacteria and allows waste milk to be stored at ambient temperatures for several days without refrigeration.

Regardless of the applied technological processing method, an additional concern with feeding waste milk to calves is the presence of residues from antimicrobial agents, which may increase the risk of antibiotic resistance in the gastrointestinal microflora^14^ and inhibit the establishment of microbial communities pertinent to the gastrointestinal tract (GIT) of neonate calves. Notably, the concentration of residual antibiotics in waste milk cannot be reduced either by pasteurisation or acidification^15^. However, few studies have demonstrated the impact on the gastrointestinal microbial community of feeding calves processed waste milk.

Therefore, the current study aimed to assess the effects of feeding dairy calves waste milk (no treatment or subjected to pasteurisation or acidification) on the digesta and mucosa-associated bacteria throughout the GIT using the Illumina HiSeq (NEB, USA) sequencing technique.

## Results

### Diet-induced changes in the bacterial composition of the rumen mucosa and digesta samples

The number of standardised operational taxonomic units (OTUs) in the digesta of calves fed pasteurised waste milk (PWM) and untreated waste milk (UWM) was significantly higher than that in calves fed acidified waste milk (AWM) and untreated bulk milk (UBM, control group) (Table 1). Microbial richness was estimated by the Chao1 index, and the value was similar among the different treatments. A comparison of the biodiversity among the dietary groups did not identify differences in the Shannon index in the bacteria associated with either the mucosa or digesta except in the UWM and UBM groups (Table 1), where the Shannon index was higher in both the rumen mucosa and digesta of the UWM calves relative to the UBM calves (Table 1).

**Figure 1:**
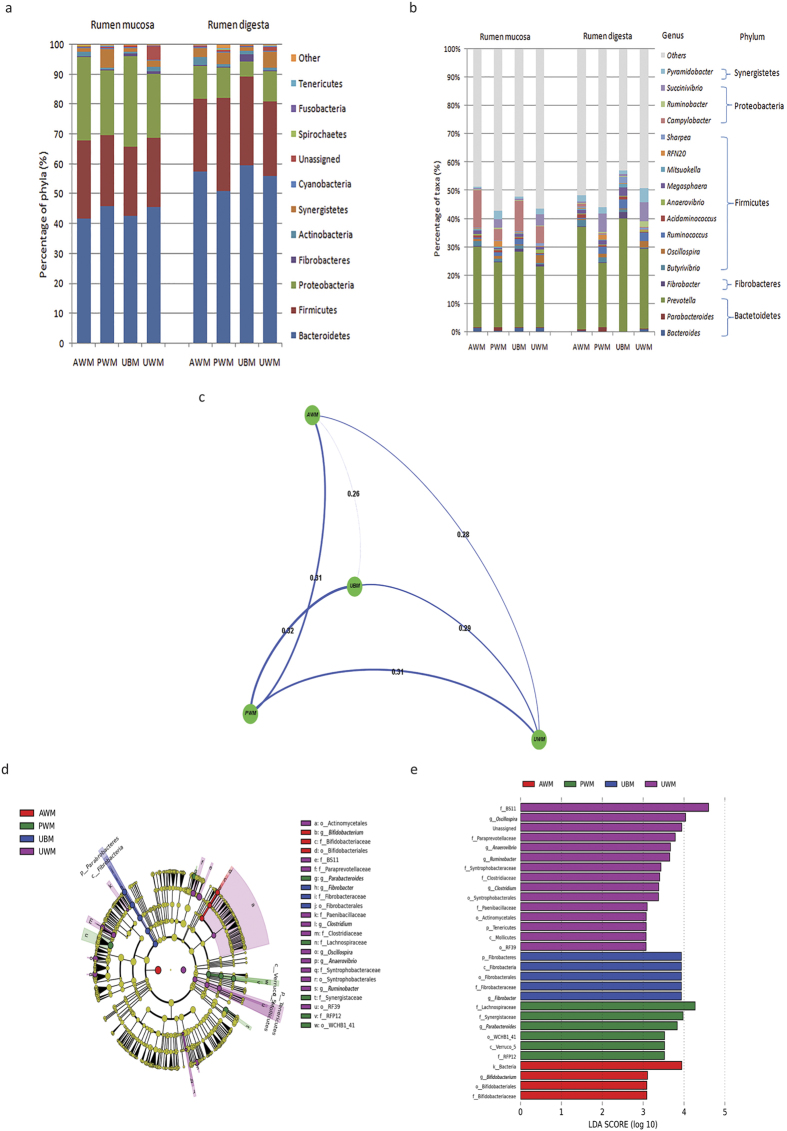
Diet-induced changes in bacterial composition based on UniFrac distances and LEfSe in the rumen. (**a**) Average relative abundances of the predominant rumen mucosa- and digesta-associated bacteria; (**b**) comparison of the predominant genera in the rumen mucosa and digesta samples (relative abundance >1% in at least one dietary group); (**c**) UniFrac distance between the groups in the rumen; (**d**) histogram of the LDA scores calculated for differentially abundant features in the rumen at the genus level among dietary groups (only the genera LDA scores above 3 are shown); and (**e**) taxonomic cladograms reporting the different taxa abundances in the rumen among the dietary groups. LEfSe, linear discriminant analysis (LDA) effect size; UBM, calves fed untreated bulk milk (control group); AWM, calves fed acidified waste milk; PWM, calves fed pasteurised waste milk; UWM, calves fed untreated waste milk.

Bacteroidetes, Firmicutes, and Proteobacteria were detected as the dominant phyla, regardless of the sampling site (mucosa or digesta) or treatment (Fig. 1a). Among the dietary treatments, the phylum Bacteroidetes was the most abundant phylum in both the mucosa and digesta bacterial communities, followed by Firmicutes and Proteobacteria, except in the mucosa samples of the AWM and UBM calves, which showed that Proteobacteria was the second most abundant phylum (Fig. 1a).

The relative abundances of the major genera in the rumen mucosa and digesta samples (relative abundance >1% in at least one dietary group) are shown in Fig. 1b. The results revealed that the predominant genera in the mucosa and digesta included *Bacteroides, Parabacteroides, Prevotella, Fibrobacter, Butyrivibrio, Oscillospira, Ruminococcus, Acidaminococcus, Anaerovibrio, Megasphaera, Mitsuokella, RFN20, Sharpea, Campylobacter, Ruminobacter, Succinivibrio*, and *Pyramidobacter*. The remaining sequences (approximately 53.6% of all sequences) were from less-predominant genera in all dietary groups or were not identified at the genus level. At the genus level, *Prevotella* was predominant among the mucosa-associated and digesta-associated bacteria in all of the dietary groups except the AWM and UBM calves, which showed a predominance of *Campylobacter* in the mucosa-associated bacteria (Fig. 1b). The relative abundances of the mucosa-associated *Fibrobacter* (q = 0.04), digesta-associated *Fibrobacter* (q = 0.03)*, Megasphaera* (q = 0.07), and *Mitsuokella* (q = 0.02) in the AWM calves were significantly lower than the abundances in the control calves (Supplementary Table S1). The relative abundance of the mucosa-associated *Parabacteroides* (q = 0.04) in the PWM calves was significantly higher than that in the control calves, whereas the levels of mucosa-associated *Fibrobacter* (q = 0.04) and digesta-associated *Fibrobacter* (q = 0.03) and *Mitsuokella* (q = 0.02) were significantly lower in the PWM calves than in the control calves (Supplementary Table S1). Our results indicated that the relative abundance of the digesta-associated *Oscillospira* (q = 0.04) in the UWM calves was significantly higher than that in the control calves, whereas the levels of digesta-associated *Fibrobacter* (q = 0.03), *Megasphaera* (q = 0.07), and *Mitsuokella* (q = 0.02) were significantly lower in the UWM calves than in the control calves (Supplementary Table S1).

Spearman’s correlation coefficients based on the UniFrac distance, which indicate the correlations among the bacterial communities in the rumen of the dietary groups, are shown in Fig. 1c. The UniFrac distance of the bacterial communities in the rumen was similar among the calves fed different types of milk. Furthermore, to detect the specific bacteria associated with each dietary group, the linear discriminant analysis (LDA) effect size (LEfSe) value was used to determine the taxonomic biomarkers. The LEfSe identified 7 bacterial genera showing biologically significant differences (Fig. 1d) and 30 differentially abundant taxonomic clades with a LDA score higher than 3.0 (Fig. 1e) among the dietary groups. At the phylum level, the abundance of Fibrobacteres was the highest in the rumen of the calves fed UBM compared with the other three treatments. However, the abundance of Tenericutes was highest in the rumen of the calves fed UWM.

### Diet-induced changes in the bacterial composition of the caecum mucosa and digesta samples

The number of standardised OTUs and alpha diversity indices (Chao1 and Shannon indices) in the colon mucosa and digesta samples are shown in Table 1. Significant differences were not observed in the number of standardised OTUs or Chao1 and Shannon indices among the dietary groups in the caecum mucosa and digesta samples except for the UWM calves (Table 1), which showed significantly higher standardised OTUs in the mucosa samples compared with the PWM and UBM calves (Table 1).

**Figure 2:**
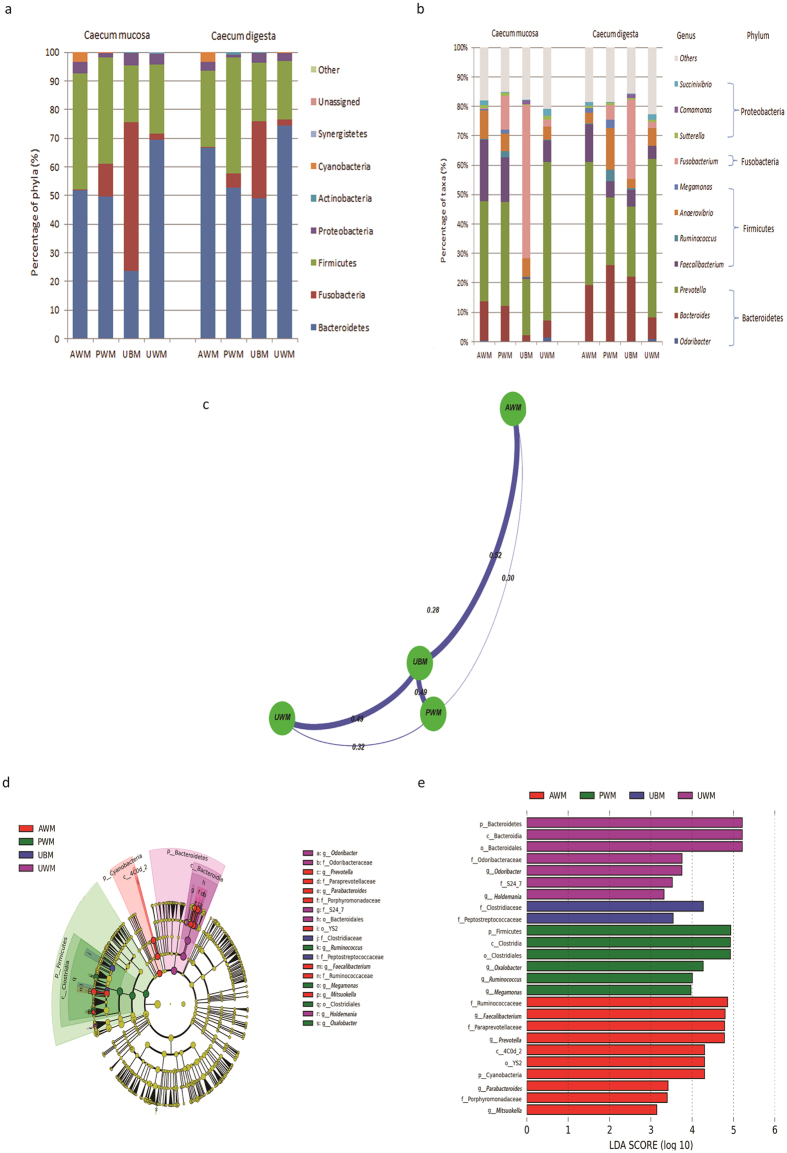
Diet-induced changes in bacterial composition based on UniFrac distances and LEfSe in the caecum. (**a**) Average relative abundances of the predominant caecum mucosa- and digesta-associated bacteria; (**b**) comparison of the predominant genera in caecum mucosa and digesta samples (relative abundance >1% in at least one dietary group); (**c**) UniFrac distance between groups in the caecum; (**d**) histogram of the LDA scores calculated for differentially abundant features in the caecum at the genus level among the dietary groups (only the genera LDA scores above 3 are shown); and (**e**) taxonomic cladograms reporting the different taxa abundances in the caecum among the dietary groups. LEfSe, linear discriminant analysis (LDA) effect size; UBM, calves fed untreated bulk milk (control group); AWM, calves fed acidified waste milk; PWM, calves fed pasteurised waste milk; UWM, calves fed untreated waste milk.

The dominant bacterial phyla in the mucosa and digesta samples of the UWM, PWM, and AWM calves were Bacteroidetes and Firmicutes, whereas the predominant phylum in the digesta samples of the UBM calves was Fusobacteria. Among the mucosa samples of the dietary groups, the maximum number of phyla was found in the UWM calves (16 phyla), followed by the UBM (15 phyla), PWM (14 phyla), and AWM calves (13 phyla). For the digesta samples, the maximum number of phyla was found in the UBM calves (13 phyla), followed by the AWM (11 phyla), PWM (10 phyla), and UWM calves (9 phyla). A comparison among the dietary groups indicated that the phylum Bacteroidetes was dominant in both the mucosa and digesta samples, except in the UBM calves, which showed a predominance of Fusobacteria in the mucosa samples. Firmicutes was the second most abundant phylum in all the dietary groups except in the calves fed UBM (Fig. 2a).

The dominant genera (relative abundance >1% in at least one diet group) in both the mucosa and digesta samples of the caecum included *Bacteroides, Odoribacter, Prevotella, Faecalibacterium, Ruminococcus, Anaerovibrio, Megamonas, Fusobacterium, Sutterella, Comamonas*, and *Succinivibrio* (Fig. 2b). At the genus level, *Prevotella* was predominant in the mucosa- and digesta-associated bacteria in all the dietary groups, except the UBM group, which showed a predominance of *Fusobacterium* (Fig. 2b). The relative abundance of the mucosa-associated *Megamonas* (q = 0.03) in the PWM calves was significantly higher than that in the control calves (Supplementary Table S2).

The UniFrac distance of the bacterial communities in the caecum between the treatment group calves and control group calves was considerably higher than that of the bacterial communities between treatment groups (Fig. 2c). The LEfSe identified 9 bacterial genera showing biologically consistent and statistically significant differences (Fig. 2d) and 25 differentially abundant taxonomic clades with a LDA score higher than 3.0 (Fig. 2e) in the different dietary groups. At the phylum level, the abundance of Cyanobacteria was highest in the caecum of the calves fed AWM among the four treatments, whereas the abundance of Firmicutes was highest in the caecum of the calves fed PWM. In addition, the caecum of the calves fed UWM contained the highest abundance of Bacteroidetes.

### Diet-induced changes in the bacterial composition of the colon mucosa and digesta samples

The number of standardised OTUs and alpha diversity indices (Chao1 and Shannon indices) among the dietary groups in the colon mucosa and digesta samples are shown in Table 1. Significant differences were not observed among the mucosa and digesta samples of the calves compared with standardised OTUs and alpha diversity indices.

**Figure 3:**
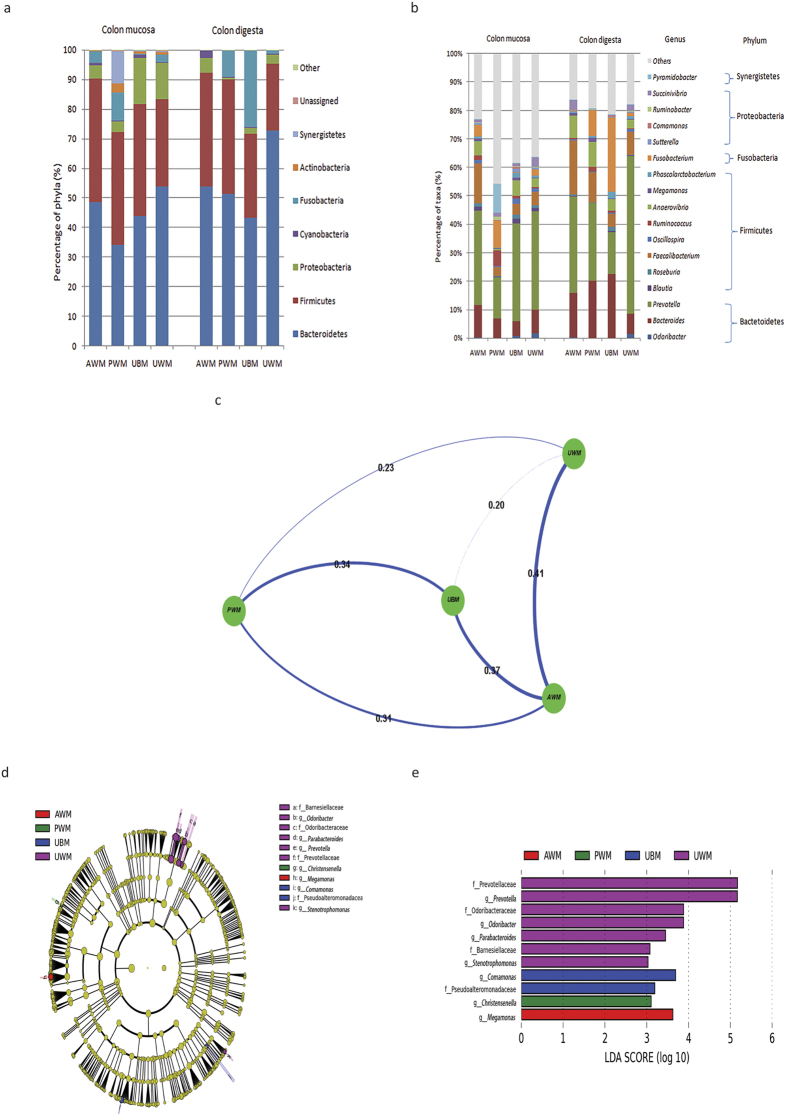
Diet-induced changes in bacterial composition based on UniFrac distances and LEfSe in the colon. (**a**) Average relative abundances of the predominant colon mucosa- and digesta-associated bacteria; (**b**) comparison of the predominant genera in colon mucosa and digesta samples (relative abundance >1% in at least one dietary group); (**c**) UniFrac distance between groups in the colon; (**d**) histogram of the LDA scores calculated for differentially abundant features in the colon at the genus level among the dietary groups (only the genera LDA scores above 3 are shown); and (**e**) taxonomic cladograms reporting the different taxa abundances in the colon among the dietary groups. LEfSe, linear discriminant analysis (LDA) effect size; UBM, calves fed untreated bulk milk (control group); AWM, calves fed acidified waste milk; PWM, calves fed pasteurised waste milk; UWM, calves fed untreated waste milk.

Bacteroidetes and Firmicutes were the dominant phyla among the mucosa- and digesta-associated bacteria in the caecum of the PWM and AWM calves, whereas Proteobacteria was the most abundant phyla among the mucosa- and digesta-associated bacteria in the caecum of the UWM and UBM calves. Among the mucosa samples of the dietary groups, the maximum number of phyla was found in the UWM calves (23 phyla), followed by the UBM (22 phyla), PWM (19 phyla), and AWM (19 phyla) calves. In the digesta samples, the maximum number of phyla was found in the PWM calves (12 phyla), followed by the UBM (10 phyla), UWM (9 phyla), and AWM calves (9 phyla). Our results showed that the phylum Bacteroidetes was dominant in both the mucosa and digesta bacterial communities except in the PWM calves, which showed a predominance of Firmicutes in the mucosa samples (Fig. 3a).

The relative abundances of the major genera in the colon mucosa and digesta samples are shown in Fig. 3b. The results revealed that the predominant genera in both the mucosa and digesta samples included *Prevotella, Bacteroides, Faecalibacterium, Succinivibrio, Anaerovibrio, Fusobacterium, Odoribacter, Blautia, Oscillospira, Roseburia, Phascolarctobacterium, Sutterella, Ruminococcus, Megamonas, Comamonas, Pyramidobacter*, and *Ruminobacter* (Fig. 3b). At the genus level, *Prevotella* was dominant among the mucosa-associated bacteria in the UWM and UBM calves, whereas *Prevotella* and *Pyramidobacter* were both dominant in the PWM calves and *Prevotella, Bacteroides*, and *Faecalibacterium* were the most abundant genera in the AWM calves (Fig. 3b). At the genus level, *Prevotella, Faecalibacterium*, and *Bacteroides* were the primary genera among the digesta-associated bacteria in the PWM and AWM calves; *Prevotella, Fusobacterium*, and *Bacteroides* were dominant among the digesta-associated bacteria in the UBM calves; and *Prevotella* was the only dominant genus in the UWM calves. The relative abundances of the mucosa-associated *Blautia* (q = 0.03) and *Phascolarctobacterium* (q = 0.03) were significantly lower in the PWM calves than in the control calves (Supplementary Table S3). The results showed that the relative abundance of the mucosa- and digesta-associated *Odoribacter* (q = 0.03 and q < 0.01, respectively) was significantly higher in the UWM calves compared with the other groups, and the relative abundance of the digesta-associated *Prevotella* (q = 0.04) was higher in the UWM calves than in the PWM and UBM calves (p < 0.05) (Supplementary Table S3).

The UniFrac distance of the bacterial communities in the colon between the UWM and UBM calves was lowest among the treatment groups when compared with the control group (Fig. 3c). Furthermore, the LEfSe identified 7 bacterial genera that showed biologically consistent differences (Fig. 3d) and 11 differentially abundant taxonomic clades with a LDA score higher than 3.0 (Fig. 3e) in the different dietary groups.

### Diet-induced changes in the bacterial composition of the rectal digesta samples

Significant differences were not observed in the number of standardised OTUs or the Chao1 and Shannon indices among the dietary groups in the rectal digesta samples (Table 1).

**Figure 4:**
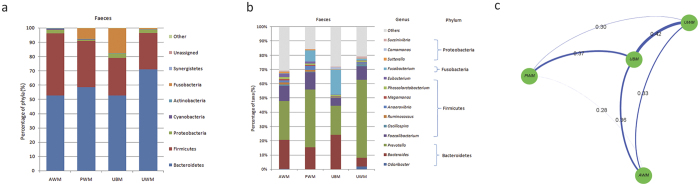
Diet-induced changes in bacterial composition based on UniFrac distances and LEfSe in the faeces. (**a**) Average relative abundances of the predominant faeces mucosa- and digesta-associated bacteria; (**b**) comparison of the predominant genera in faeces mucosa and digesta samples (relative abundance >1% in at least one dietary group); and (**c**) UniFrac distance between groups in the faeces. UBM, calves fed untreated bulk milk (control group); AWM, calves fed acidified waste milk; PWM, calves fed pasteurised waste milk; UWM, calves fed untreated waste milk.

Bacteroidetes and Firmicutes were the dominant phyla in the rectal digesta samples from all of the dietary groups. The maximum number of phyla was found in the UWM calves (13 phyla), followed by the AWM (10 phyla), UBM (10 phyla) and PWM (9 phyla) calves. Among all the dietary groups, the phylum Bacteroidetes was dominant in the rectal bacterial community (Fig. 4a).

The dominant genera in the digesta samples of the rectum were *Odoribacter, Bacteroides, Prevotella, Faecalibacterium, Oscillospira, Ruminococcus, Anaerovibrio, Megamonas, Phascolarctobacterium, Eubacterium, Fusobacterium, Sutterella, Comamonas*, and *Succinivibrio*. At the genus level of the faeces-associated bacteria, *Prevotella, Bacteroides*, and *Faecalibacterium* were predominant in the PWM and AWM calves, *Prevotella*, Bacteroides, and *Fusobacterium* were the dominant genera in the UBM calves, and *Prevotella* was dominant in the UWM calves (Fig. 4b). Specifically, the richness of *Odoribacter* (q = 0.01) in the UWM calves was significantly higher than that in the other dietary groups (Supplementary Table S4). The UniFrac distance of the bacterial communities in the faeces between the control and the other dietary group was similar (Fig. 4c). Furthermore, clades were not detected by the LDA of the LEfSe for each dietary group.

### Diet-induced changes in the metabolic pathways of the gastrointestinal microbiome

**Figure 5:**
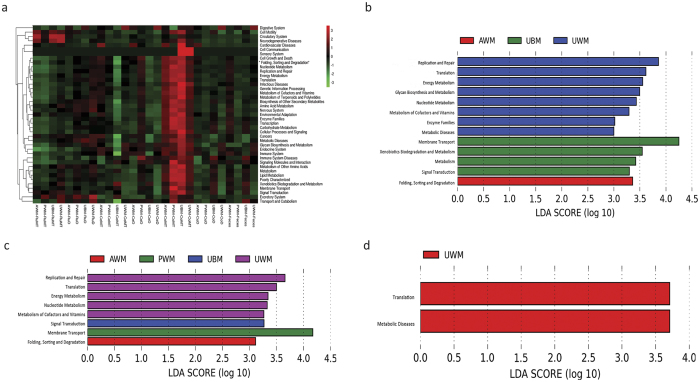
Comparison of KEGG pathways predicted by PICRUSt according to diet. (**a**) Heatmap of 41 second-level classification KEGG pathways identified in the gut samples. Histogram of the LDA scores calculated for the differentially abundant features in the caecum (**b**), colon (**c**), and faeces (**d**) for second-level classification KEGG pathways among the dietary groups (only the LDA scores above 3 are shown). LDA, linear discriminant analysis; UBM, calves fed untreated bulk milk (control group); AWM, calves fed acidified waste milk; PWM, calves fed pasteurised waste milk; UWM, calves fed untreated waste milk.

A total of 299 third-level classification Kyoto Encyclopedia of Genes and Genomes (KEGG) pathways were verified based on the structure of the gastrointestinal microbiota established using PICRUSt, which is a predictive expeditionary tool. Our study identified 41 second-level classification KEGG pathways in the rumen, caecum, colon (mucosa or digesta), and faeces samples by PICRUSt (Fig. 5a). The statistically significant KEGG pathways of each group were identified by the LEfSe. We found that 11 second-level classification KEGG pathways significantly differed among the caecum of the calves in the 4 dietary groups (Fig. 5b) and 8 second-level classification KEGG pathways were markedly different in the colon (Fig. 5c). Furthermore, 2 second-level classification KEGG pathways differed considerably in the faeces of the 4 groups (Fig. 5d). Among these groups, the calves fed UWM exhibited the most differential second-level classification KEGG pathways, whereas certain metabolic biomarkers were consistent across the different GITs. Finally, differential KEGG pathways were not detected in the rumen of calves in the different dietary groups.

## Discussion

In this study, we characterised the bacterial taxonomic compositions and phylogenetic distributions in the mucosa and digesta in different parts of the GIT of pre-weaned dairy calves that received different milk regimens. Significant differences in bacterial richness and diversity were indicated among the dietary groups (Table 1), suggesting that the diet had a prominent effect on the microbial community composition of pre-weaned dairy calves. Similar results have been observed in mice, and diet was found to exert a dominant effect in shaping the inter-individual variations in host-associated microbial communities^16^.

The current results demonstrated that the microbial community structures occupying the GITs of the pre-weaned dairy cattle were influenced by the four types of milk. Although the gastrointestinal bacterial communities of the pre-weaned dairy calves were all dominated by the genus *Prevotella*, the predominant genera varied among the four dietary groups and across the different parts of the GIT. In addition, the four dietary groups influenced the mucosa- or digesta-associated bacterial diversity. Specifically, our study showed that feeding UWM to calves led to an increase of 2 phyla and 10 genera compared with the calves fed the other three types of milk. These phyla and genera included Tenericutes, *Clostridium, Oscillospira, Anaerovibrio,* and *Ruminobacter* in the rumen; Bacteroidetes, *Odoribacter* and *Holdemania* in the caecum; and *Prevotella, Odoribacter, Parabacteroides,* and *Stenotrophomonas* in the colon. However, compared with the calves fed the other three types of milk, the calves fed AWM exhibited the highest abundances of Cyanobacteria, *Faecalibacterium, Prevotella, Parabacteroides,* and *Mitsuokella* in the caecum; *Bifidobacterium* in the rumen; and *Megamonas* in the colon. The calves fed PWM had the highest abundances of Firmicutesin, *Oxalobacter, Ruminococcus,* and *Megamonas* in the caecum; *Parabacteroides* in the rumen; and *Christensenella* in the colon compared with the calves fed the other three types of dietary milk. The calves fed UBM showed the highest abundances of Fibrobacteres and *Fibrobacter* in the rumen and *Comamonas* in the colon compared with the calves fed the other three types of dietary milk. Finally, the increased expression of genes related to metabolic diseases in the caecum and faeces of the calves fed UWM indicated that UWM is not a suitable liquid diet for pre-weaned dairy calves.

In the rumen, the bacterial compositions and distributions were similar among the 4 diets at the phylum level, except in the UBM calves, which showed a predominance of Fibrobacteres, and in in the UWM calves, which showed a predominance of Tenericutes (p < 0.05; Fig. 1d and 1e). However, the relative abundances of these two phyla were both low and did not exceed 3% in all calves. At the genus level, 7 dominant genera were detected among the 4 dietary groups. In addition, the AWM calves exhibited a higher relative abundance of mucosa- and digesta-associated *Bifidobacterium* (Fig. 1e), which was likely induced by the greater amounts of formic acid and the lower rumen pH associated with this diet^17^,^18^. This probiotic bacteria genus has been shown to enhance the hydrolysis of volatile solids^19^, and it has the ability to hydrogenate linoleic acid^20^. In addition, we found that feeding calves PWM elevated the abundance of the genus *Parabacteroides*, which is responsible for protein and polysaccharide degradation^21^. Such an elevated abundance of *Parabacteroides* may have been related to the relatively larger quantities of amino acids and monosaccharides observed in the rumen of the PWM-fed dairy calves. Moreover, feeding calves with UBM elevated the relative abundance in the rumen (both mucosa and digesta) of the genus *Fibrobacter,* which is responsible for the degradation of dietary fibre and polysaccharides, and this result was likely related to the lack of antibiotics in the UBM^22^,^23^. Therefore, more volatile fatty acids and monosaccharides may have been produced in the rumen of dairy calves fed UBM. The present study also indicated that feeding dairy calves UWM increased the abundance of *Anaerovibrio, Oscillospira, Clostridium*, and *Ruminobacter,* which was likely caused by the potential presence of pathogenic bacteria in the UWM. Certain members of the genus *Anaerovibrio* are able to utilise glycerol^24^ and have been associated with propionate and butyrate production in the rumen^24^,^25^. Previous studies revealed that squid ink polysaccharide and ciprofloxacin treatment were correlated with a reduced proportion of the genus *Oscillospira* in the gut^26^,^27^. Hence, rumen concentrations of propionate and butyrate may be increased by feeding dairy calves UWM. Certain members of the genus *Clostridium* represent opportunistic pathogens that cause gut inflammation and are generally associated with intestinal dysbiosis^28^. Therefore, feeding pre-weaned calves UWM may increase the chance of gastrointestinal disorders. However, the increased relative abundance of genus *Ruminobacter* in the calves fed UWM could not be explained and requires further study.

In the caecum, the bacterial compositions and distributions were similar among the dietary groups at the phylum level, although in the UBM calves, a greater abundance of Cyanobacteria was observed; in in the PWM calves, a greater abundance of Firmicutes was observed; and in the UWM calves, a greater abundance of Bacteroidetes was observed (Fig. 2d and 2e). At the genus level, 9 significantly different genera were detected among the dietary groups, and the results indicated that the abundances of the genera *Prevotella, Parabacteroides, Faecalibacterium*, and *Mitsuokella* were remarkably enhanced in the caecum (both mucosa and digesta) of the AWM calves compared with the other calves. The abundance of *Prevotella* in the caecum of the AWM calves may have been caused by the acid content and the concomitant low pH^22^,^24^. A previous report indicated that the relative abundance of *Prevotella* in the caecum tended to increase linearly in rabbits that drank acidified drinking water^29^. Consistent with our findings, adding barley acidified with citric acid increased the *in vitro* relative abundance of *Prevotella*^30^, which is a polysaccharide-degrading bacterial genus^23^. Together, these findings suggest that adding organic acids to a liquid diet and maintaining a low pH may represent a feasible strategy for rearing pre-weaned dairy calves. Comparatively, the proportion of the genus *Faecalibacterium* was increased in the caecum of the AWM calves, which is similar to the findings for piglets fed fermented liquid feed with a low pH^31^. However, the reason for the increased relative abundances of the genera *Parabacteroides* and *Mitsuokella* in the caecum of dairy calves fed AWM remains unclear and deserves further investigation. The results also indicated that feeding calves PWM elevated the relative abundance of *Ruminococcus, Megamonas*, and *Oxalobacter* in the caecum (both mucosa and digesta). Notably, certain members of *Ruminococcus* produce short chain fatty acids, which are important sources of energy for ruminants^32^. In addition, the genus *Oxalobacter* has an important symbiotic relationship with its hosts because it can regulate oxalic acid homeostasis^33^. These findings implied that feeding dairy calves PWM may exert beneficial effects on calf health by elevating the abundance of beneficial bacteria in the caecum. However, the results indicated that feeding UWM to pre-weaned dairy calves increased the relative abundance of *Holdemania* and *Odoribacter*, which may have been related to the presence of these bacteria in the UWM. A previous study found that the genus Holdemania is associated with lean cattle^34^, and certain members of this genus have been found to increase the activity of proton pump inhibitors^35^. The genus *Odoribacter* may be associated with certain diseases and the presence of stress^36^,^37^. Altogether, these results indicated that UWM is not a proper diet for pre-weaned dairy calves.

In the colon, the bacteria compositions and distributions were similar among the calves at the phylum level (Fig. 3a and 3b); however, significant differences were observed in 7 bacterial genera among the different dietary treatments (Fig. 3d and 3e). The results suggest that AWM enriched *Megamonas* in the colon, which is a type of butyrate-producing bacteria associated with enzymes involved in propionate production pathways^38^. Reports have shown that the genus *Megamonas* is significantly increased in healthy individuals compared with diseased individuals^39^,^40^. These results suggest that feeding AWM to calves may enhance the overall health conditions of dairy calves. In addition, our data revealed that *Christensenella* was enriched in the colon of the PWM calves, but the underlying mechanism was unclear. A recent study suggested that the presence in the gut of *Christensenella*, which is a low abundance (less than 0.001%) and highly heritable (transmissible from parent to offspring) bacterial genus, decreased the body weight gain of obese mice, thereby confirming that a low abundance of microbiota can impact host physiology^41^. However, such a claim requires further validation to determine the functional mechanisms underlying the reduced body weight gain in the presence of *Christensenella*. The present study also demonstrated that UBM heightened the relative abundance of the genus *Comamonas,* which is related to the degradation of steroids and various aromatic acids^42^,^43^. These previous studies implied that the hydrolytic and fermentative functions of UBM in the colon may have been enhanced. Furthermore, the results of the present study revealed that *Prevotella, Parabacteroides, Stenotrophomonas*, and *Odoribacter* were enriched in the UWM dietary group, and this enrichment may have been induced by potential pathogenic bacteria in the UWM. Previous reports have shown that the genus *Prevotella* is increased in the colon of goats fed high grain diets^44^. Therefore, the higher abundance of this genus in the UWM-fed calves was likely related to higher starch contents in the colon. Furthermore, the genus *Stenotrophomonas* is an organophosphorus degrading organism^45^, and certain members of this genus act as the infectious pathogens of Crohn’s disease^46^. Consequently, UWM is not suitable as a diet for pre-weaned dairy calves because it may harbour certain pathogens and would likely lead to an increased incidence of calf GIT diseases. However, in the current study, the mechanisms underlying the elevated abundance of *Parabacteroides* and *Odoribacter* in the colon of dairy calves fed UWM were not identified. Finally, the dietary treatments did not have an effect on the rectal bacterial communities associated with digesta and mucosa at any phylogenetic level.

In the current study, we also identified the potential functions of the pre-weaned dairy calf gastrointestinal microbiome in the mucosa and digesta samples using PICRUSt to predict the metabolic pathways based on the 16S rRNA gene sequences^47^. We found that the most abundant second-level metabolic functional classifications were membrane transport, carbohydrate metabolism, amino acid metabolism, replication and repair, and translation and energy metabolism, and these findings were consistent with the results of a previous study on adult dairy cows^48^. Notably, more second-level metabolic pathways were identified in the colon than in the rumen (Fig. 5a). The present study also observed marked differences in the bacterial second-level metabolic functions in the GIT components of the calves in the different dietary groups (Fig. 5b, 5c and 5d) except in the rumen. For example, the increased expression of genes related to folding, sorting, and degradation was observed in the caecum and colon of calves fed AWM, whereas the increased expression of genes related to membrane transport was observed in the colon of calves fed PWM. In addition, the increased expression of genes related to signal transduction was observed in the caecum and colon of calves fed UBM, and a greater number of genes related to translation were identified in the caecum, colon, and faeces of calves fed UWM. In particular, an increased abundance of genes associated with metabolic diseases was observed in the caecum and faeces of calves fed UWM. These findings indicated that UWM as a diet may promote the development of disease in pre-weaned dairy calves, and this conclusion is consistent with previous results obtained for bacteria (i.e., *Clostridium, Odoribacter* and *Stenotrophom*). Further experiments are planned to verify the relationships of the listed metabolic functions with the ingestion of the four types of milk.

## Methods

### Experimental animals

A total of 84 calves (Holstein; males; single birth) from multifarious cows were used in the study. The experiment was conducted on a commercial dairy farm (>10,000 heads) in Anhui Province, China. The calves were randomly assigned to one of the four treatment groups directly after birth, and they were housed for the first 6 weeks of life in individual hutches that included outdoor pens with straw bedding. New straw was added weekly. The management and feeding of the calves in the four experimental groups were identical except for the type of milk. All of the experimental protocols were approved by the Committee on Animal Care of China Agricultural University, and all of the procedures were performed following the guidelines of the China Council on Animal Care.

### Experimental diets

Each calf was fed 4 litres of thawed frozen colostrum (IgG > 50 mg/ml) using a nipple bottle within 1 h after birth. Thereafter, the calves were fed UBM (control group), AWM, PWM, or UWM. The calves were fed twice daily with equal volumes of milk via nipple buckets, and the amounts corresponded to a total volume of 322 litres within the experimental period between day 1 to day 42 of life. Bulk milk was obtained from the milking line twice daily, and waste milk was collected twice daily into a specific tank. Pasteurisation (high temperature over a short time) was performed by heating the milk at 73 °C for 15 s. Acidification was performed by adding formic acid (30 ml of 9.8% formic acid to 1 litre of milk) to waste milk at 5 °C and placing the mixture at ambient temperature for at least 24 h. The milk was fed at 38–40 °C. The nipple buckets were cleaned every day with a brush using hot tap water and a commercial detergent and then rinsed with clear water. A pelleted calf starter (19.9% crude protein, 2.4% crude fat, 14.9% crude fibre, 87.6% neutral detergent fibre, 11.3% acid detergent fibre, and 18.9 mega joule metabolic energy/kg; Cargill, Anhui, China) was offered from postnatal day 4 until day 42. The amounts of starter and milk intake were recorded daily for each calf.

Waste milk on the farm was composed of low-quality colostrum, transitional milk, milk from cows administered veterinary drugs for the treatment of mastitis or other diseases, and milk with high somatic cell counts. The antimicrobial agents included amoxicillin, benzylpenicillin, cefalexin, ceftiofur, cloxacillin, dihydrostreptomycin, flunixin meglumine, gentamycin, kanamycin, meloxicam, nafcillin, oxytetracycline, sodium salicylate, and tulathromycin.

### Sample collection

On postnatal day 21, 3 calves from each group were randomly euthanised, and mucosal tissue and digesta samples were collected from the rumen, caecum, colon, and faeces within 20 min after euthanisation^49^. The mucosal tissue samples were cut into small pieces and washed with sterile phosphate-buffered saline (PBS, pH 7.0) to remove the digesta. The digesta samples were collected from the mucosal tissue samples. The rumen dorsal sac, rumen ventral sac, rumen digesta, rumen fluid, rumen residue, caecum mucosal tissue, caecum digesta, colon mucosal tissue, colon digesta, and faeces samples were collected in sterile tubes and stored in liquid nitrogen until further analysis.

### DNA extraction

The genomic DNA of all samples was extracted from 1 g total ruminal, caecal, colonic, and rectal contents or 1 ml ruminal fluid using a QIAGEN DNA Extraction Kit^TM^ (Qiagen, Valencia, CA, USA) according to the manufacturer’s protocol using a repeated bead beating method, which was followed by phenol-chloroform extraction. The DNA was re-suspended after precipitation with ethanol. The DNA quality was assessed based on the absorbance ratios of 260/280 nm and 260/230 nm using a NanoDrop ND-1000 Spectrophotometer (Wilmington, DE, USA).

### Sequencing

Sequencing libraries were generated using the NEB Next Ultra DNA sample preparation kit (New England Biolabs, Ipswich, MA, USA) following the standard Illumina sample-preparation protocol, and index codes were added. The quality of the library was assessed using a Qubit 2.0 Fluorometer (Life Technologies, Grand Island, NY) and an Agilent Bioanalyzer 2100 system (Agilent Technologies, Palo Alto, CA). The library was sequenced on an Illumina HiSeq platform, and single 150 bp × 2 paired-end reads were generated.

### Quality control of raw data and data processing

The raw data quality was controlled using FASTQC^50^, and reads with a quality score higher than 30 were retained for further analysis. Paired-end reads from the original DNA fragments were merged using FLASH (version 1.2.7)^51^ and assigned to each sample according to their unique barcodes. Concatenated sequences were detected using USEARCH (v8.0)^52^ and subsequently filtered out. Sequences analyses were performed using the QIIME pipeline (version 1.5.0)^53^. Generated sequences were distributed into different samples based on barcodes, and the OTUs were defined by clustering sequences together with a 97% identity cut-off setting by the UCLUST software^54^ after removing the barcode and primers. The RDP classifier^55^ was used for the taxonomic classification of the generated OTUs using the Greengenes database^56^. Standardised OTU documents were used to analyse the species and diversity indices to ensure the comparability of the species diversity between the samples. The threshold for the number of standardised sequences was set at 100,000 sequences. In case the number of sequences in one sample was the smallest among all of the samples, then that value was used as the threshold. Alpha diversity indices, including Chao 1 and Shannon, were calculated using the QIIME pipeline (version 1.5.0)^53^. Rarefaction curves were generated based on the observed species and the PD whole tree. The LEfSe^57^ was used to determine the taxonomic biomarkers.

### Statistical analysis

Statistical analyses were conducted using R software (version 3.2.2) (https://www.r-project.org/). All of the parameters were represented as the average and standard deviation of 4 groups. The statistical significance of the taxonomic groups was determined by a one-way analysis of variance (ANOVA). Fisher’s protected least significant difference (LSD) test was used for multiple treatment comparisons of the taxonomic groups. A p-value < 0.05 was considered to indicate statistical significance. In addition, the p-value from the multiple comparison analyses within the ruminal microbial community was adjusted by the FDR using the *p. adjust* package in R. The relative abundance analysis was assessed by the LEfSe using the results of the Kruskal-Wallis and Wilcoxon tests, and the threshold on the logarithmic LDA score was 3.0. Spearman’s correlation coefficient was calculated to identify correlations between the diet and biomarkers and the bacterial abundance. The original 16S rRNA Illumina sequencing reads (n = 119) were deposited at the National Center for Biotechnology Information (NCBI) Sequence Read Archive database under the accession IDs SRR3501074, SRR3501133, SRR3501118, SRR3501069, and PRJNA321239.

## Additional Information

**Accession codes:** The original 16S rRNA Illumina sequencing reads (n = 119) have been deposited at the National Center for Biotechnology Information Sequence Read Archives database under the accession IDs SRR3501074, SRR3501133, SRR3501118, SRR3501069, and PRJNA321239.

**How to cite this article**: Deng, Y. F. *et al*. Influence of dairy by-product waste milk on the microbiomes of different gastrointestinal tract components in pre-weaned dairy calves. *Sci. Rep.* **7**, 42689; doi: 10.1038/srep42689 (2017).

**Publisher's note:** Springer Nature remains neutral with regard to jurisdictional claims in published maps and institutional affiliations.

## Supplementary Material

Supplementary Information

## Figures and Tables

**Table 1:** Operational taxonomic units (OTUs) and alpha diversity (Chao1 and Shannon indices) for the diet groups.

		Treatment^1^				SEM^2^	P-value
Region		AWM	PWM	UBM	UWM		
Rumen mucosa	OTUs	1564.00	1935.50	1462.50	1819.33	199.379	0.331
	Chao1 index	2128.55	1618.84	1717.53	2409.66	339.732	0.346
	Shannon index	5.54^ab^	4.67^ab^	4.24^b^	5.69^a^	0.454	0.103
Rumen digesta	OTUs	1455.78^b^	1891.00^a^	1346.67^b^	1953.44^a^	129.773	0.004
	Chao1 index	1642.94	2385.56	1479.96	1865.90	341.914	0.282
	Shannon index	5.09^ab^	5.14^ab^	4.13^b^	5.43^a^	0.415	0.158
Caecum mucosa	OTUs	832.00^ab^	670.33^b^	647.67^b^	979.33^a^	79.357	0.058
	Chao1 index	2629.59	2725.59	2671.01	2922.79	514.121	0.978
	Shannon index	5.76	5.33	6.05	5.82	0.423	0.692
Caecum digesta	OTUs	806.67	651.33	659.33	904.67	106.886	0.336
	Chao1 index	1715.13	1211.14	1081.42	1721.59	219.060	0.151
	Shannon index	5.06	4.73	3.83	5.22	0.413	0.159
Colon mucosa	OTUs	1276.67	1860.67	1625.67	1779.33	397.125	0.742
	Chao1 index	2545.38	3669.42	2581.65	3221.72	329.653	0.117
	Shannon index	5.51	6.41	5.50	5.91	0.295	0.174
Colon digesta	OTUs	774.00	636.33	675.00	884.33	139.340	0.614
	Chao1 index	2371.25	3301.03	2282.15	2982.24	360.626	0.218
	Shannon index	5.26	6.52	5.45	6.24	0.446	0.213
Faeces	OTUs	931.00	616.00	626.67	977.00	145.985	0.234
	Chao1 index	2877.62	2978.49	2449.02	3643.36	355.297	0.203
	Shannon index	5.83	5.72	5.50	6.39	0.414	0.513

Note. OTUs represent the number of standardised OTUs; the Chao1 index is used to estimate the microbial richness; and the Shannon index is used to assess the biodiversity.

^1^UBM, calves fed untreated bulk milk (control group); AWM, calves fed acidified waste milk; PWM, calves fed pasteurised waste milk; UWM, calves fed untreated waste milk.

^a,b^Means within a row with different superscripts differ (p < 0.05).

^2^SEM, standard error of the means.
