# Supplementary material for: Influence of dairy by-product waste milk on the microbiomes of different gastrointestinal tract components in pre-weaned dairy calves
Source: Sci Rep. 2017 Mar 10;7:42689. doi: 10.1038/srep42689 (PMC5345013; doi:10.1038/srep42689)
Supplement: Supplementary Information [file srep42689-s1.docx]

**Supplementary Figures and Tables**

**Influences of dairy by-product** **waste-milk on the microbiomes of different gastrointestinal tract components of pre-weaned dairy calves**

Y. F. Deng^1,^†, Y. J. Wang^1,^†, Y. Zou^1^, A. Azarfar^2^, X. L. Wei^3^, S. K. Ji^1^, J. Zhang^1^, Z. H. Wu^1^, S. X. Wang^1^, S. Z. Dong^1^, Y. Xu^4^, D. F. Shao^1^, J. X. Xiao^1^, K. L. Yang^5,^*, Z. J. Cao^1,^*&S. L. Li^1,^*

^1^State Key Laboratory of Animal Nutrition, Beijing Engineering Technology Research Center of Raw Milk Quality and Safety Control, College of Animal Science and Technology, China Agricultural University, Beijing 100193, P. R. China

^2^Department of Animal Science, Faculty of Agriculture, Lorestan University, PO Box 465, Khorramabad, Iran

^3^Sichuan Animal Science Academy, Animal Breeding and Genetics key Laboratory of Sichuan Province, Chengdu 610066, P. R. China

^4^Beijing Computing Center, Beijing 100094, P. R. China

^5^ College of Animal Science, Xinjiang Agricultural University, Wulumuqi 830052, P. R. China

†These authors contributed equally to this work

*Co-corresponding authors. Correspondence and requests for materials should be addressed to S. L. L. (email: lisheng0677@163.com).

**Supplementary Table S1**

**Supplementary Table S2**

**Supplementary Table S3**

**Supplementary Table S4**

**Table S1**. Relative abundance of mucosa- and digesta-associated diet-induced predominant genera in the rumen (%).

| *Phylum* | *Genus* | Rumen——Treatment | | | | | | | | | | | |
| --- | --- | --- | --- | --- | --- | --- | --- | --- | --- | --- | --- | --- | --- |
|  |  | Mucosal Tissue | | | | SEM^2^ | p-value | Digesta | | | | SEM | p-value |
|  |  | AWM^1^ | PWM^1^ | UBM^1^ | UWM^1^ |  |  | AWM | PWM | UBM | UWM |  |  |
| Bacteroidetes | *Bacteroides* | 1.68 | 0.48 | 1.70 | 1.54 | 0.006 | 0.647 | 0.60^ab^ | 0.29^b^ | 0.28^b^ | 0.89^a^ | 0.002 | 0.085 |
|  | *Parabacteroides* | 0.07^b^ | 1.31^a^ | 0.05^b^ | 0.20^b^ | 0.003 | 0.041 | 0.44^ab^ | 1.52^a^ | 0.01^b^ | 0.23^ab^ | 0.004 | 0.096 |
|  | *Prevotella* | 28.50 | 22.81 | 26.67 | 21.33 | 0.043 | 0.684 | 36.28^a^ | 22.70^b^ | 39.74^a^ | 28.35^ab^ | 0.040 | 0.021 |
| Fibrobacteres | *Fibrobacter* | 0.24^b^ | 0.23^b^ | 1.02^a^ | 0.83^ab^ | 0.002 | 0.041 | 0.09^b^ | 0.24^b^ | 2.31^a^ | 0.17^b^ | 0.004 | 0.001 |
| Firmicutes | *Butyrivibrio* | 1.68 | 1.01 | 1.16 | 0.56 | 0.004 | 0.635 | 2.23^a^ | 1.68^ab^ | 1.13^ab^ | 0.30^b^ | 0.005 | 0.065 |
|  | *Oscillospira* | 0.70^ab^ | 0.99^ab^ | 0.35^b^ | 2.61^a^ | 0.007 | 0.604 | 0.21^b^ | 1.33^ab^ | 0.08^b^ | 2.19^a^ | 0.004 | 0.003 |
|  | *Ruminococcus* | 0.44 | 1.29 | 1.96 | 0.52 | 0.007 | 0.635 | 0.49 | 2.33 | 3.42 | 3.21 | 0.010 | 0.179 |
|  | *Acidaminococcus* | 0.95 | 0.88 | 0.74 | 0.26 | 0.003 | 0.661 | 1.46^a^ | 0.74^ab^ | 1.12^a^ | 0.04^b^ | 0.003 | 0.036 |
|  | *Anaerovibrio* | 0.29^ab^ | 0.11^ab^ | 0.05^b^ | 1.30^a^ | 0.004 | 0.607 | 0.08^ab^ | 0.16^ab^ | 0.01^b^ | 0.64^a^ | 0.002 | 0.073 |
|  | *Megasphaera* | 1.23 | 0.60 | 1.03 | 0.97 | 0.003 | 0.661 | 1.31^b^ | 1.53^ab^ | 3.00^a^ | 0.14^b^ | 0.005 | 0.006 |
|  | *Mitsuokella* | 0.54 | 0.35 | 0.47 | 0.29 | 0.002 | 0.815 | 0.67^b^ | 0.21^b^ | 1.48^a^ | 0.03^b^ | 0.002 | 0.000 |
|  | *RFN20* | 0.21 | 2.25 | 0.05 | 0.05 | 0.007 | 0.604 | 0.05^b^ | 1.58^a^ | 0.02^b^ | 0.08^b^ | 0.005 | 0.061 |
|  | *Sharpea* | 0.49^ab^ | 0.19^b^ | 0.45^ab^ | 0.92^a^ | 0.002 | 0.635 | 0.43^b^ | 0.25^b^ | 2.13^a^ | 0.62^b^ | 0.005 | 0.044 |
| Proteobacteria | *Campylobacter* | 13.02 | 4.03 | 10.83 | 6.14 | 0.029 | 0.635 | 1.12^a^ | 0.30^b^ | 0.49^b^ | 0.29^b^ | 0.002 | 0.012 |
|  | *Ruminobacter* | 0.39 | 0.30 | 0.12 | 0.10 | 0.001 | 0.635 | 0.26 | 0.52 | 0.00 | 1.92 | 0.007 | 0.257 |
|  | *Succinivibrio* | 0.34 | 3.00 | 0.42 | 4.02 | 0.013 | 0.635 | 0.42^b^ | 6.44^ab^ | 0.62^b^ | 6.81^a^ | 0.020 | 0.044 |
| Synergistetes | *Pyramidobacter* | 0.48 | 3.00 | 0.75 | 2.01 | 0.009 | 0.635 | 2.35^ab^ | 2.35^ab^ | 1.20^b^ | 4.94^a^ | 0.010 | 0.064 |

^1^ UBM, calves fed untreated bulk milk (control group); AWM, calves fed acidified waste milk; PWM, calves fed pasteurised waste milk; UWM, calves fed untreated waste milk. a, b Means within a row with different superscripts differ (p< 0.05). ^2^ SEM, standard error of the means.

**Table S2**. Relative abundance of mucosa- and digesta-associated diet-induced predominant genera in the cecum (%).

| *Phylum* | *Genus* | Cecum——Treatment | | | | | | | | | | | |
| --- | --- | --- | --- | --- | --- | --- | --- | --- | --- | --- | --- | --- | --- |
|  |  | Mucosal Tissue | | | | SEM^2^ | p-value | Digesta | | | | SEM | p-value |
|  |  | AWM^1^ | PWM^1^ | UBM^1^ | UWM^1^ |  |  | AWM | PWM | UBM | UWM |  |  |
| Bacteroidetes | *Odoribacter* | 0.23^b^ | 0.00^b^ | 0.12^b^ | 1.32^a^ | 0.003 | 0.026 | 0.11^b^ | 0.00^b^ | 0.09^b^ | 0.94^a^ | 0.002 | 0.064 |
|  | *Bacteroides* | 13.53 | 12.23 | 2.14 | 5.78 | 0.038 | 0.188 | 19.02 | 25.97 | 21.91 | 7.30 | 0.115 | 0.701 |
|  | *Prevotella* | 34.02 | 35.35 | 18.97 | 53.97 | 0.113 | 0.264 | 41.97 | 23.01 | 23.89 | 53.96 | 0.121 | 0.282 |
| Firmicutes | *Faecalibacterium* | 20.80 | 15.08 | 0.66 | 7.37 | 0.061 | 0.182 | 12.89 | 5.57 | 5.72 | 4.25 | 0.037 | 0.391 |
|  | *Ruminococcus* | 0.27 | 1.99 | 0.21 | 0.12 | 0.007 | 0.263 | 0.23^b^ | 3.90^a^ | 0.43^b^ | 0.11^b^ | 0.010 | 0.076 |
|  | *Anaerovibrio* | 9.83 | 6.21 | 6.16 | 4.61 | 0.041 | 0.834 | 3.66^b^ | 14.24^a^ | 3.21^b^ | 6.11^ab^ | 0.030 | 0.103 |
|  | *Megamonas* | 0.52^ab^ | 1.18^a^ | 0.07^b^ | 0.06^b^ | 0.002 | 0.018 | 1.48 | 2.78 | 0.18 | 0.04 | 0.008 | 0.122 |
| Fusobacteria | *Fusobacterium* | 0.18^b^ | 11.56^ab^ | 51.99^a^ | 2.27^b^ | 0.140 | 0.096 | 0.08 | 4.84 | 26.88 | 1.95 | 0.131 | 0.479 |
| Proteobacteria | *Sutterella* | 1.05 | 1.05 | 0.44 | 1.22 | 0.003 | 0.355 | 0.64 | 1.03 | 0.37 | 0.71 | 0.002 | 0.270 |
|  | *Comamonas* | 0.00 | 0.00 | 1.23 | 0.02 | 0.006 | 0.443 | 0.00 | 0.00 | 1.29 | 0.00 | 0.006 | 0.441 |
|  | *Succinivibrio* | 1.59 | 0.23 | 0.19 | 2.27 | 0.009 | 0.331 | 1.34 | 0.05 | 0.28 | 1.92 | 0.010 | 0.531 |

^1^ UBM, calves fed untreated bulk milk (control group); AWM, calves fed acidified waste milk; PWM, calves fed pasteurised waste milk; UWM, calves fed untreated waste milk. a, b Means within a row with different superscripts differ (p< 0.05). ^2^ SEM, standard error of the means.

| *Phylum* | *Genus* | Colon——Treatment | | | | | | | | | | | |
| --- | --- | --- | --- | --- | --- | --- | --- | --- | --- | --- | --- | --- | --- |
|  |  | Mucosal Tissue | | | | SEM^2^ | p-value | Digesta | | | | SEM | p-value |
|  |  | AWM^1^ | PWM^1^ | UBM^1^ | UWM^1^ |  |  | AWM | PWM | UBM | UWM |  |  |
| Bacteroidetes | *Odoribacter* | 0.32^b^ | 0.10^b^ | 0.83^b^ | 1.76^a^ | 0.003 | 0.008 | 0.19^b^ | 0.00^b^ | 0.17^b^ | 1.43^a^ | 0.001 | 0.000 |
|  | *Bacteroides* | 11.36 | 6.82 | 5.15 | 8.25 | 0.040 | 0.729 | 15.67 | 20.29 | 22.36 | 7.25 | 0.101 | 0.731 |
|  | *Prevotella* | 33.23 | 14.50 | 34.44 | 34.57 | 0.058 | 0.107 | 34.07^ab^ | 27.39^b^ | 14.81^b^ | 55.29^a^ | 0.078 | 0.035 |
| Firmicutes | *Blautia* | 1.38^a^ | 0.38^b^ | 1.50^a^ | 1.14^a^ | 0.002 | 0.014 | 0.16 | 0.07 | 0.27 | 0.24 | 0.001 | 0.758 |
|  | *Roseburia* | 1.16 | 0.07 | 1.57 | 1.01 | 0.005 | 0.294 | 0.44 | 0.00 | 1.58 | 0.35 | 0.008 | 0.547 |
|  | *Faecalibacterium* | 13.99^a^ | 3.03^b^ | 3.75^ab^ | 4.78^ab^ | 0.031 | 0.108 | 18.98 | 10.65 | 4.46 | 7.98 | 0.067 | 0.498 |
|  | *Oscillospira* | 1.08 | 0.54 | 1.73 | 1.04 | 0.003 | 0.197 | 0.50 | 0.11 | 0.58 | 0.89 | 0.003 | 0.473 |
|  | *Ruminococcus* | 1.79^ab^ | 5.35^a^ | 1.07^ab^ | 0.59^b^ | 0.013 | 0.105 | 0.35^b^ | 1.61^a^ | 0.58^b^ | 0.18^b^ | 0.002 | 0.009 |
|  | *Anaerovibrio* | 4.82^b^ | 0.62^b^ | 5.52^b^ | 2.93^ab^ | 0.009 | 0.017 | 7.75 | 08.87 | 4.02 | 3.40 | 0.040 | 0.715 |
|  | *Megamonas* | 0.98^a^ | 0.21^b^ | 0.92^ab^ | 0.23^b^ | 0.002 | 0.062 | 0.96 | 1.38 | 0.22 | 0.11 | 0.004 | 0.156 |
|  | *Phascolarctobacterium* | 0.85^ab^ | 0.21^b^ | 1.52^a^ | 0.85^ab^ | 0.002 | 0.015 | 0.69 | 0.62 | 2.43 | 0.72 | 0.011 | 0.611 |
| Fusobacteria | *Fusobacterium* | 3.85 | 9.33 | 0.31 | 2.14 | 0.048 | 0.600 | 0.10 | 9.09 | 25.93 | 1.21 | 0.129 | 0.502 |
| Proteobacteria | *Sutterella* | 1.09^a^ | 0.40^b^ | 1.08^a^ | 0.80^ab^ | 0.002 | 0.116 | 0.77 | 0.53 | 0.24 | 0.74 | 0.002 | 0.378 |
|  | *Comamonas* | 0.00 | 0.01 | 1.13 | 0.06 | 0.005 | 0.435 | 0.00 | 0.00 | 0.58 | 0.00 | 0.003 | 0.440 |
|  | *Ruminobacter* | 0.01 | 1.22 | 0.01 | 0.01 | 0.005 | 0.339 | 0.00 | 0.00 | 0.00 | 0.00 | 0.000 | 0.219 |
|  | *Succinivibrio* | 1.16 | 1.23 | 0.96 | 3.49 | 0.011 | 0.377 | 3.28 | 0.07 | 0.39 | 2.40 | 0.017 | 0.506 |
| Synergistetes | *Pyramidobacter* | 0.03^b^ | 10.32^a^ | 0.02^b^ | 0.02^b^ | 0.028 | 0.071 | 0.00 | 0.01 | 0.00 | 0.00 | 0.000 | 0.467 |

**Table S3**. Relative abundance of mucosa- and digesta-associated diet-induced predominant genera in the colon (%).

^1^ UBM, calves fed untreated bulk milk (control group); AWM, calves fed acidified waste milk; PWM, calves fed pasteurised waste milk; UWM, calves fed untreated waste milk. a, b Means within a row with different superscripts differ (p< 0.05). ^2^ SEM, standard error of the means.

**Table S4**. Relative abundance of diet-induced predominant genera in faeces (%).

| *Phylum* | *Genus* | Faeces-Treatment | | | | | |
| --- | --- | --- | --- | --- | --- | --- | --- |
|  |  | Digesta | | | | SEM^2^ | p-value |
|  |  | AWM^1^ | PWM^1^ | UBM^1^ | UWM^1^ |  |  |
| Bacteroidetes | *Odoribacter* | 0.32^b^ | 0.00^b^ | 0.13^b^ | 2.05^a^ | 0.003 | 0.003 |
|  | *Bacteroides* | 20.24 | 15.38 | 24.08 | 6.08 | 0.101 | 0.635 |
|  | *Prevotella* | 27.28^ab^ | 40.60^ab^ | 20.27^b^ | 54.72^a^ | 0.095 | 0.128 |
| Firmicutes | *Faecalibacterium* | 10.64 | 11.98 | 5.47 | 9.46 | 0.055 | 0.849 |
|  | *Oscillospira* | 1.10 | 0.45 | 0.25 | 1.29 | 0.006 | 0.531 |
|  | *Ruminococcus* | 1.38 | 1.23 | 0.48 | 0.24 | 0.005 | 0.365 |
|  | *Anaerovibrio* | 1.29 | 2.77 | 0.21 | 1.36 | 0.008 | 0.264 |
|  | *Megamonas* | 1.11 | 0.86 | 0.33 | 0.16 | 0.003 | 0.213 |
|  | *Phascolarctobacterium* | 1.37 | 0.84 | 1.01 | 0.84 | 0.006 | 0.898 |
|  | *Eubacterium* | 2.51 | 1.42 | 0.13 | 0.31 | 0.009 | 0.263 |
| Fusobacteria | *Fusobacterium* | 0.13 | 7.81 | 17.58 | 0.98 | 0.091 | 0.537 |
| Proteobacteria | *Sutterella* | 1.44^a^ | 1.00^ab^ | 0.31^b^ | 0.44^ab^ | 0.003 | 0.124 |
|  | *Comamonas* | 0.00 | 0.00 | 1.22 | 0.00 | 0.006 | 0.441 |
|  | *Succinivibrio* | 0.47 | 0.11 | 0.50 | 1.15 | 0.005 | 0.475 |

^1^ UBM, calves fed untreated bulk milk (control group); AWM, calves fed acidified waste milk; PWM, calves fed pasteurised waste milk; UWM, calves fed untreated waste milk. a, b Means within a row with different superscripts differ (p< 0.05). ^2^ SEM, standard error of the means.
